# Trees follow rhythms: diel dynamics of nonstructural carbohydrates are influenced by environment, taxonomy, and functional traits

**DOI:** 10.1093/plphys/kiag153

**Published:** 2026-03-16

**Authors:** Yuzhen Fan, Laura Fernández-de-Uña

**Affiliations:** Assistant Features Editor, Plant Physiology, American Society of Plant Biologists; Division of Plant Sciences, Research School of Biology, Australian National University, Canberra, ACT 2601, Australia; Assistant Features Editor, Plant Physiology, American Society of Plant Biologists; Department of Plant Biology and Soil Sciences, Universidade de Vigo, Ourense 32004, Spain

Plants use nonstructural carbohydrates (NSCs; ie those not composing plant tissues) to maintain many key physiological processes, such as metabolite synthesis, respiration, growth, osmotic regulation, and defense (Hartmann and Trumbore 2016). Plants produce glucose during photosynthesis, which is then transformed into other metabolic compounds. In woody species, photosynthetic tissues are mainly located in the leaves; therefore, assimilated carbon, in the form of NSCs, needs to be translocated to other tree organs (ie branches, stem, and roots). Photosynthesis is influenced, directly or via stomatal regulation, by a number of factors, including radiation, temperature, vapor pressure deficit (VPD; which indicates how dry the air is), and water availability. As a result, photosynthesis is generally reduced under environmental stress or low radiation. Metabolic and physiological processes consuming carbohydrates, such as osmoregulation (Tomasella et al. 2021), respiration (Crous et al. 2022), and growth (Rathgeber et al. 2022), are also affected by environmental conditions.

Under optimal growth conditions, photosynthetic rates peak at midday (Gersony et al. 2020), when radiation is maximal. While part of the synthesized glucose is readily used for leaf metabolic processes and osmotic regulation, high midday carbon assimilation rates can cause a surplus of glucose and other derived sugars (Gersony et al. 2020). These excess sugars are converted into reserve compounds, including sucrose and starch. Starch is insoluble, thus remaining in the leaf for longer-term storage. By contrast, sucrose can be transported through the phloem to other tree organs, where it is either used for cellular processes or converted into and stored as starch. During periods of absent or limited photo-assimilation, starch is hydrolyzed to glucose and stored sugars are consumed to meet the tree's metabolic needs (Hartmann and Trumbore 2016). The feedback between carbon supply and demand thus leads to seasonal and daily cycles in the concentrations of soluble sugars and starch across tree organs. Understanding NSC fluctuations and their coupling across organs within the tree is key to better characterizing carbon allocation, transport, and storage within trees and ultimately to improve models of tree and ecosystem carbon balance.

Recently in *Plant Physiology*, Li et al. (2026) measured high-resolution diel changes in soluble sugar and starch concentrations in leaves and branches of 15 tree species at 4 environmentally distinct sites. The authors found that diel variation in NSC concentrations was driven not only by temporal factors but also by functional traits, species identity, and environmental conditions. Diel soluble sugar and starch dynamics were highly synchronized between leaves and branches, suggesting fast NSC translocation from the former to the latter without phloem loading limitations (Gersony et al. 2020). NSC concentrations showed consistent diel variation in both leaves and branches, peaking in the late afternoon and declining to a minimum by dawn (Fig. 1). However, this variation slightly differed between soluble sugars and starch, with starch peaking earlier and reaching a greater magnitude than soluble sugars. Such discrepancy likely reflects the distinct roles of these 2 types of carbohydrates. Soluble sugars are maintained above a critical threshold to support osmotic regulation and metabolic processes, thus fluctuating less over a day (Sala et al. 2012). By contrast, starch serves as a transient reserve that can be fully remobilized and is regulated daily by a circadian clock (Smith and Stitt 2007). Overall, NSC levels measured between 9 AM and 11 AM closely matched the daily average, providing an optimal window for standardized sampling.

**Figure 1:**
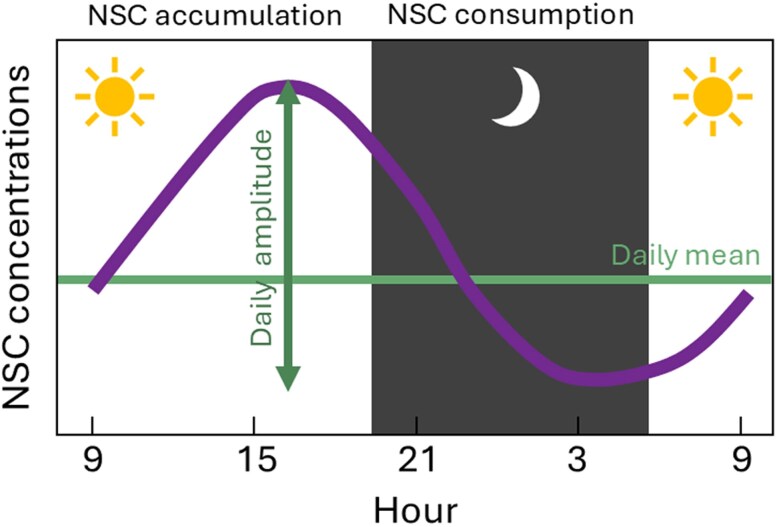
Schematic diagram of diel dynamics in NSC concentrations in trees observed by Li et al. (2026). The purple curve represents the diel trajectory of NSC concentration in a tree organ over 24 h. The green horizontal line indicates the daily mean of NSC concentrations, while the green double-headed arrow denotes the magnitude of diel variation in NSC.

Further analysis of tree traits across species revealed that wood density was correlated with average daily NSC concentrations, whereas tree diameter was associated with the magnitude of diel variation in NSC (Li et al. 2026). Previous studies have shown that trees with denser wood maintain greater long-term NSC storage, while larger and older trees (ie with greater diameter) buffer diel fluctuations in NSC more effectively, resulting in fewer changes in NSC concentrations over a day (Tixier et al. 2018). Li et al. (2026) also highlighted that the diel-driven variation in NSC concentrations is explained more by environmental conditions than by species identity or functional traits. Among the environmental factors examined, VPD was identified as the primary driver of diel variation in NSC concentrations. For example, sites with higher VPD variability had lower mean NSC concentrations in leaves and branches, whereas sites with higher mean VPD showed greater diel NSC fluctuations in branches. This finding suggests that in warmer and drier future climates where VPD is high, trees may have lower carbon and NSC storage, increasing the risk of carbon starvation and tree mortality (Sevanto et al. 2014).

This study advanced our understanding of how daily NSC concentrations are regulated across tree species with contrasting functional traits and diverse climates of origin. The results highlight the importance of incorporating tissue-specific diel feedback between soluble sugar and starch pools into models that currently assume fixed carbon allocation. Accounting for short-term environmental variability is crucial to predict carbon dynamics in trees. Hence, future studies should further explore diel carbon fluctuations along the growing season and across tree organs to better account for the influence of different environmental conditions and organ phenological stages on the observed NSC dynamics.

**Recent related articles in *Plant Physiology*:**

- Fan et al. (2024) measured diel variation in different metabolites, including NSCs, across 8 C_3_ and C_4_ grasses, and showed that leaf dark respiration is governed by multiple concurrent CO_2_-producing and O_2_-consuming metabolic pathways, including those involved in sugar interconversion.
- Siqueira et al. (2025) found that watering timing affects NSC accumulation, vegetative growth, and reproductive yield in tomato via circadian and flowering gene expression.
- Vincent et al. (2025) reviewed the balance between photosynthetic activity and transport capacity in conifers and *Citrus* spp. and how this balance influences NSC accumulation and phloem transport.

## Data Availability

No new data were generated or analyzed in support of this article.

## References

[kiag153-B1] Crous KY, Uddling J, De Kauwe MG. 2022. Temperature responses of photosynthesis and respiration in evergreen trees from boreal to tropical latitudes. New Phytol. 234:353–374. 10.1111/nph.17951. 35007351 PMC9994441

[kiag153-B2] Fan Y et al 2024. Variation in leaf dark respiration among C3 and C4 grasses is associated with use of different substrates. Plant Physiol. 195:1475–1490. 10.1093/plphys/kiae064. 38324704 PMC11142371

[kiag153-B3] Gersony JT et al 2020. Leaf carbon export and nonstructural carbohydrates in relation to diurnal water dynamics in mature oak trees. Plant Physiol. 183:1612–1621. 10.1104/pp.20.00426. 32471810 PMC7401141

[kiag153-B4] Hartmann H, Trumbore S. 2016. Understanding the roles of nonstructural carbohydrates in forest trees—from what we can measure to what we want to know. New Phytol. 211:386–403. 10.1111/nph.13955. 27061438

[kiag153-B5] Li W, Pu Y, Li F, Jiang Y. 2026. High-resolution diel dynamics of non-structural carbohydrates in trees reveal organ-level coordination and trait–environment coupling. Plant Physiol. 200:kiag123. 10.1093/plphys/kiag123. 41787725

[kiag153-B6] Rathgeber CBK et al 2022. Anatomical, developmental and physiological bases of tree-ring formation in relation to environmental factors. In: Siegwolf RTW, Brooks JR, Roden J, Saurer M, editors. Stable isotopes in tree rings: inferring physiological, climatic and environmental responses. Springer International Publishing. p. 61–99.

[kiag153-B7] Sala A, Woodruff DR, Meinzer FC. 2012. Carbon dynamics in trees: feast or famine? Tree Physiol. 32:764–775. 10.1093/treephys/tpr143. 22302370

[kiag153-B8] Sevanto S, McDowell NG, Dickman LT, Pangle R, Pockman WT. 2014. How do trees die? A test of the hydraulic failure and carbon starvation hypotheses. Plant Cell Environ. 37:153–161. 10.1111/pce.12141. 23730972 PMC4280888

[kiag153-B9] Siqueira JA et al 2025. Differential impact of dawn and dusk watering on tomato metabolism and biomass allocation. Plant Physiol. 198:e11270. 10.1093/plphys/kiaf227. 40473243

[kiag153-B10] Smith AM, Stitt M. 2007. Coordination of carbon supply and plant growth. Plant Cell Environ. 30:1126–1149. 10.1111/j.1365-3040.2007.01708.x. 17661751

[kiag153-B11] Tixier A, Orozco J, Roxas AA, Earles JM, Zwieniecki MA. 2018. Diurnal variation in nonstructural carbohydrate storage in trees: remobilization and vertical mixing. Plant Physiol. 178:1602–1613. 10.1104/pp.18.00923. 30366979 PMC6288742

[kiag153-B12] Tomasella M et al 2021. Shade-induced reduction of stem nonstructural carbohydrates increases xylem vulnerability to embolism and impedes hydraulic recovery in *Populus nigra*. New Phytol. 231:108–121. 10.1111/nph.17384. 33811346 PMC9290559

[kiag153-B13] Vincent C, Hussain SB, Losada JM, Liesche J. 2025. Source–sink transport as a constraint on photosynthesis and a driver of ecophysiological patterns. Plant Physiol. 199:503. 10.1093/plphys/kiaf531. 41118528

